# Monitoring of Antimicrobial Resistance Genes and Susceptibility Profiles in Bacterial Isolates From Animal‐Origin Meat

**DOI:** 10.1111/1750-3841.70719

**Published:** 2025-11-23

**Authors:** André Schenkel Dedecek, Lavinia Arend, Margareth Leonor Penkal, Salesia Maria Prodocimo Moscardi, Felipe Francisco Tuon

**Affiliations:** ^1^ Laboratory of Emerging Infectious Diseases, School of Medicine Pontifícia Universidade Católica do Paraná Curitiba Brazil; ^2^ Laboratório Central do Estado do Paraná São Jose dos Pinhais Brazil; ^3^ Vigilância Sanitária de Alimentos da Secretaria de Saúde do Estado do Paraná Curitiba Brazil

**Keywords:** antimicrobial resistance, extended‐spectrum β‐lactamases, foodborne bacteria, meat safety, one health

## Abstract

The excessive use of antibiotics in animal production has contributed to the global spread of antimicrobial resistance (AMR), posing a significant public health threat. To evaluate the antimicrobial susceptibility profiles and detect resistance genes in bacterial isolates from chicken, pork, and fish meat samples collected in the State of Paraná, Brazil, between 2017 and 2023. Meat samples (n = 418) were tested for *Escherichia coli* and *Salmonella* spp., while *Klebsiella* spp. (n = 265) and *Pseudomonas* spp. (n = 262) were included from 2019 onward. Bacterial isolation followed ISO 17604:2015 standards with MALDI‐TOF identification. Susceptibility testing was performed using the Kirby‐Bauer disk diffusion method. Detection of resistance genes (bla_TEM, bla_SHV, bla_CTX‐M, bla_KPC, bla_NDM, bla_OXA‐48, bla_VIM, bla_SPM, and mcr‐1) was conducted by real‐time PCR. Multidrug resistance (MDR) was widespread, particularly in *Salmonella spp*. and *Pseudomonas spp*., followed by *Klebsiella spp*. and *E. coli*. Extended‐spectrum β‐lactamase (ESBL) genes were frequent in *E. coli* and *Klebsiella spp*., while plasmid‐mediated colistin resistance (*mcr‐1*) and carbapenemase (*bla*KPC) were also identified. Resistance to third‐generation cephalosporins and ampicillin was especially high. Sampling across years and animal species may not fully reflect farm‐level resistance. The absence of molecular typing limited the ability to track clonal spread of resistant strains. Widespread AMR in foodborne bacteria was observed, with resistance levels often exceeding those reported in European monitoring. These findings underscore the need for strengthened surveillance and coordinated actions across animal and human health sectors.

## 1 Introduction

The use of antibiotics has been the predominant strategy for combating bacterial infections, with many available molecules developed between 1930 and 1960. However, the indiscriminate use of these compounds and the lack of new antibiotics are crucial factors driving the spread of antimicrobial resistance (AMR) (Samtiya et al. 2022). Concern over this issue led to the first discussions on regulating antibiotic use in animal health as early as 1969 (Lees et al. 2021). In recent years, the global demand for animal protein has risen significantly and continues to grow (Mulchandani et al. 2023). With this increase in demand and production, the use of antimicrobials in intensive animal farming systems has also expanded, both for the treatment of infections and as growth promoters in feed (Tiseo et al. 2020).

Veterinary use of antibiotics now surpasses human consumption in many parts of the world (Van Boeckel et al. 2017). This widespread use contributes to the emergence and dissemination of AMR mechanisms, affecting the environment, foodborne microorganisms, and ultimately human health (Schar et al. 2020). Previous studies estimate that approximately 73% of all antimicrobials sold globally are used in animal production, highlighting the urgent need for monitoring resistance linked to veterinary antibiotic use (Van Boeckel et al. 2017). In 2017, Brazil ranked second globally in veterinary antimicrobial consumption, accounting for 7.9% of global use—exceeding the combined use of France, Germany, Spain, the United Kingdom, and Poland (Tiseo et al. 2020).

Over the past two decades, there has been an expansion in networks for monitoring antimicrobial usage and resistance, driven partly by initiatives of the World Health Organization (WHO) in 2013 and 2014 (Chandra Deb et al. 2023). These efforts culminated in a 2016 United Nations report that described the AMR crisis and outlined global recommendations. It is estimated that, without action, AMR could lead to 10 million deaths annually by 2050, with a projected economic loss of USD 100 trillion due to decreased productivity and increased healthcare costs (Review on Antimicrobial Resistance 2016).

In Brazil, governmental decrees were published beginning in 2016 to establish committees tasked with developing the National Action Plan for the Prevention and Control of Antimicrobial Resistance (PAN‐BR), and in 2017, the Action Plan for Health Surveillance in Antimicrobial Resistance (PAN‐VISA) was launched (BRASIL ANVISA 2021). As part of these strategies, the PAMVet PR (Veterinary Drug Monitoring Program of Paraná) was restructured in 2017 to guide public health decisions. The program incorporated antimicrobial susceptibility profiling, resistance gene detection, and monitoring of antimicrobial residues in commercially available food products.

The research initiative described here was prompted by the detection of polymyxin‐resistant bacteria in samples from non‐hospitalized patients (community‐acquired infections), animals, and the environment in Brazil (Rau et al. 2020). In response, PAMVet PR began investigating the presence of polymyxin resistance genes (mcr‐1) in *E. coli* and *Salmonella* spp. from poultry samples, recognizing the clinical importance of polymyxins as last‐resort antibiotics against multidrug‐resistant pathogens.

Given that Brazil is one of the largest global producers of animal protein—with Paraná State leading national production of chicken, pork, and tilapia—and considering the insufficient regulatory control over veterinary antibiotic use, monitoring the emergence and spread of AMR genes from animal sources becomes imperative. Food products of animal origin contaminated with resistant bacteria represent a significant risk for the transfer of resistance genes to human microbiota and pathogenic organisms. Nevertheless, monitoring of AMR linked to veterinary sources remains less developed compared to surveillance efforts focused on human‐origin resistant microorganisms (Tiseo et al. 2020).

The aim of this study was to evaluate the antimicrobial susceptibility profiles and detect circulating resistance genes in bacterial isolates obtained from chicken, pork, and fish meat samples sold in the State of Paraná, Brazil, between 2017 and 2023, with comparisons with European data.

## 2 Methods

### 2.1 Sample Collection

**FIGURE 1:**
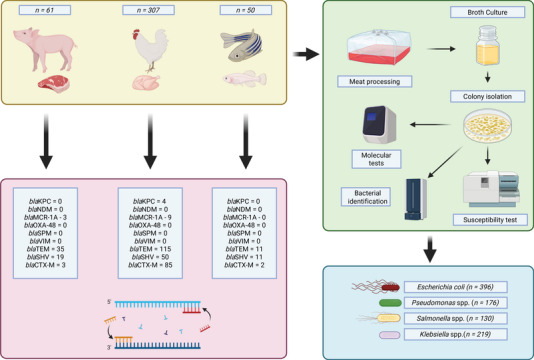
Workflow of bacterial isolation and characterization from meat samples of animal origin (chicken, pork, and fish). The process includes meat processing, broth culture, colony isolation, bacterial identification, antimicrobial susceptibility testing, and molecular detection of resistance genes. The table summarizes the distribution of key resistance genes among *E. coli* (n = 396), *Klebsiella* spp. (n = 219), *Pseudomonas* spp. (n = 176), and *Salmonella* spp. (n = 130), including carbapenemases (bla_KPC, bla_NDM, bla_OXA‐48, bla_SPM, bla_VIM), colistin resistance (bla_MCR‐1A), and extended‐spectrum β‐lactamases (bla_TEM, bla_SHV, bla_CTX‐M).

Chicken, pork, and fish meat samples were collected from 2017 to 2023 by the Municipal Health Surveillance Agencies of the State of Paraná, Brazil. Collections followed the procedures outlined in Guide 19, Version 2 (December 23, 2019) of the Brazilian Health Regulatory Agency (ANVISA) for Sampling, Packaging, Transportation, Reception, and Destination of Samples for Laboratory Analysis within the National Health Surveillance System. Figure 1 resumes some steps of the process.

Initial analyses in 2017 focused exclusively on isolating *E. coli* and *Salmonella* spp. from poultry samples to investigate the presence of *mcr‐1* genes encoding resistance to polymyxins B and E. Although the use of polymyxins as growth promoters in animal feed was prohibited in 2016, the sale of existing stocks was allowed under transitional legislation. In late 2018, the project team expanded the scope to include broader resistance gene detection and antimicrobial susceptibility testing in isolates collected from 2019 to 2023. Additional bacterial targets, *Klebsiella* spp. and *Pseudomonas* spp., were incorporated due to their clinical relevance: *Klebsiella* spp. for its multidrug resistance profiles and mobile genetic elements, and *Pseudomonas* spp. as an important environmental bacterium associated with biofilms and water systems (e.g., slaughterhouse sprinklers). Priority was given to sampling meat products, whether portioned or whole, in their original factory packaging, to minimize contamination risks from the retail environment.

### 2.2 Bacterial Isolation

Isolation procedures were adapted from ISO 17604:2015 with modifications by the Central Laboratory of Paraná (LACEN/PR). Each meat sample was placed in a sterile bag and immersed in sufficient buffered peptone water to cover the sample. After sealing the bag without air bubbles, manual agitation and massaging ensured thorough contact with the diluent. The wash fluid was then used for inoculation into enrichment broths and selective culture media targeting the bacteria of interest. Bacterial species identification was performed using MALDI‐TOF mass spectrometry (VITEK MS, bioMérieux).

### 2.3 Preparation of Bacterial Inoculum

Isolated strains were reactivated in Brain Heart Infusion (BHI) broth and plated onto MacConkey agar to obtain fresh, pure cultures. Bacterial suspensions were prepared by transferring 1 to 3 colonies into 3 mL of sterile 0.9% saline. The turbidity was adjusted to match a 0.5 McFarland standard (approximately 1.5 × 10⁸ CFU/mL) using a turbidimeter. This suspension was used for antimicrobial susceptibility testing and DNA extraction.

### 2.4 Antimicrobial Susceptibility Testing

The Kirby‐Bauer disk diffusion method was employed on Mueller‐Hinton agar plates (150 mm diameter). Bacterial suspensions were evenly spread across plates using a sterile swab in three directions. Selected antimicrobial disks (imipenem, ceftazidime, cefoxitin, amoxicillin/clavulanate, cefepime, cefotaxime, ciprofloxacin, chloramphenicol, ampicillin, gentamicin, tetracycline, azithromycin, sulfamethoxazole/trimethoprim, and tigecycline) were then placed onto the surface. Plates were incubated at 35°C for 18–24 hours. After incubation, inhibition zone diameters were measured in millimeters and interpreted following the EUCAST guidelines, categorizing isolates as susceptible, intermediate, or resistant. Quality control was performed using *E. coli* ATCC 25922 (EUCAST 2021).

### 2.5 Minimum Inhibitory Concentration (MIC) for Colistin

MIC determinations for colistin were performed using the broth microdilution method. Serial dilutions of colistin were prepared in cation‐adjusted Mueller‐Hinton broth. Wells were inoculated with 50 µL of bacterial suspension standardized to 7.5 × 10⁵ CFU/mL. Plates were incubated at 35 ± 2°C for 16 – 20 h. MIC readings were taken as the lowest concentration inhibiting visible bacterial growth. Results were interpreted according to EUCAST criteria (Berkhout et al. 2015).

### 2.6 DNA Extraction

DNA was extracted using the crude extract method. Briefly, 500 µL of bacterial suspension was transferred to 1.5 mL microtubes, heated at 97°C for 5 min (Loccus, Cotia, Brazil), and then stored at −80°C until further analysis by qPCR (Arend et al. 2015).

### 2.7 Real‐Time PCR (qPCR)

qPCR assays were performed using TaqMan Fast Advanced Master Mix (Applied Biosystems by Thermo Fisher Scientific, Waltham, MA, USA) with primer and probe concentrations of 20 µM and 10 µM, respectively (Table 1). Reactions (11 µL volume) were conducted in 96‐well optical plates sealed with optical adhesive film and run on a QuantStudio 5 Real‐Time PCR System (Applied Biosystems, Waltham, MA, USA). Automatic threshold settings were used for result interpretation, with positive and negative controls included in all assays. qPCR cycling conditions included decontamination at 50°C for 2 min, Taq activation at 95°C for 10 min, amplification (40 cycles) at 95°C for 15 s and 60°C for 1 min, and detection at 60°C for 1 min (Arend et al. 2023).

**TABLE 1:** Primers and probes used for gene detection in animal sample meat for carbapenemases, polymyxin resistance and extended‐spectrum beta‐lactamases.

Target gene	Forward primer (5’–3’)	Reverse primer (5’–3’)	Probe (5’‐3’)
*bla*KPC	GGC CGC CGT GCA ATA C	GCC GCC CAA CTC CTT CA	FAM‐TGA TAA CGC CGC CGA TTT GT‐BHQ
*bla*NDM	GAC CGC CCA GAT CCT CAA	CGC GAC CGG CAG GTT	VIC‐TGG ATC AAG CAG GAG AT‐BHQ
*bla*MCR‐1A	GCA GCA TAC TTC TGT GTG GTA C	ACA AAG CCG AGA TTG TCC GCG	CY5‐GAC CGC GAC CGC CAAT CTT ACC‐BHQ
*bla*OXA‐48	TCC GGC CAC GGA GCA AAT CAG	GAT GTG GGC ATA TCC ATA TTC ATC GCA	CY5‐CTG GCT GCG CTC CGA TAC GTG TAA CTT ATT G‐BHQ2
*bla*SPM	AGG CAA GGT CTT CTC GTT TT	GCA TCT CCC AGA TAA CCA AGT T	FAM‐TGC CCC GAT AAT GTC GTC GTA TAT T ‐BHQ1
*bla*VIM	GAT GAG TTG CTT TTG ATT GAT ACA GC	CCG ACK CGR TCG TCA T	CY5‐TGC CGG AGA TTG ARA AGC AAA TTG GA‐BHQ1
*bla*TEM	GCA TCT TAC GGA TGG CAT GA	GTC CTC CGA TCG TTG TCA GAA	FAM‐CAG TGC TGC CAT AAC CAT GAG TGA‐BHQ1
*bla*SHV	TCC CAT GAT GAG CAC CTT TAA A	TCC TGC TGG CGA TAG TGG AT	VIC‐TGC CGG TGA CGA ACA GCT GGA G‐BBQ650
*bla*CTX‐A	CGG GCR ATG GCG CAR AC	TGC RCC GGT SGT ATT GCC	CY5‐CCA RCG GGC GCA GYT GGT GAC‐BHQ1
*bla*CTX‐B	ACC GAG CCS ACG CTC AA	CCG CTG CCG GTT TTA TC	CY5‐CCC GCG YGA TAC CAC CAC GC‐BHQ1

## 3 Results

### 3.1 General Data

Between 2017 and 2023, a total of 418 samples were analyzed for the presence of *E. coli* and *Salmonella* spp., including 307 chicken samples, 61 pork samples, and 50 fish samples. Due to differences in the initiation dates of isolation for *Klebsiella* spp. and *Pseudomonas* spp. (as described in Materials and Methods), the number of samples tested for these bacteria varied slightly. For *Klebsiella* spp., 265 samples were analyzed (154 chicken, 61 pork, and 50 fish), and for *Pseudomonas* spp., 262 samples were analyzed (153 chicken, 61 pork, and 48 fish).

As shown in Table 2, among the 418 samples tested for *E. coli*, 396 were positive, resulting in an overall isolation rate of 94.74%. When broken down by sample type, the isolation rates were 98.7% (n = 303) for chicken, 86.9% (n = 53) for pork, and 80% (n = 40) for fish. For *Salmonella* spp., the overall isolation rate was 31.1% (n = 130), with rates of 36.16% (n = 111) for chicken, 21.3% (n = 13) for pork, and 12% (n = 6) for fish.

**TABLE 2:** Detection rates of *E. coli*, *Klebsiella* spp., *Pseudomonas* spp., and *Salmonella* spp. in samples of chicken, pork, and fish. The table presents the total number and percentage of positive samples for each bacterium, as well as the detection of resistance genes, including carbapenemases (KPC, NDM, SPM, VIM, OXA‐48), extended‐spectrum β‐lactamases (ESBL: TEM, CTX‐M, SHV), and the colistin resistance gene (MCR). Results are expressed both in absolute numbers and as percentages of the total samples tested for each food category.

Bacteria		Total		Chicken		Pork		Fish	
*Escherichia coli*		Samples (n = 418)	%	Samples (n = 307)	%	Samples (n = 61)	%	Samples (n = 50)	%
	Detected	396	95%	303	99%	53	87%	40	80%
Carbapenemases		4	1%	4	1%	0	0%	0	0%
	KPC	4	1%	4	1%	0	0%	0	0%
	NDM	0	0%	0	0%	0	0%	0	0%
	SPM	0	0%	0	0%	0	0%	0	0%
	VIM	0	0%	0	0%	0	0%	0	0%
	OXA‐48	0	0%	0	0%	0	0%	0	0%
ESBL									
	TEM	112	27%	85	28%	23	38%	4	8%
	CTX‐M	58	14%	55	18%	2	3%	1	2%
	SHV	17	4%	13	4%	2	3%	2	4%
MCR		10	2%	9	3%	1	2%	0	0%
*Klebsiella* spp.		Samples (n = 265)	%	Samples (n = 154)	%	Samples (n = 61)	%	Samples (n = 50)	%
	Detected	219	83%	125	81%	52	85%	42	84%
Carbapenemases		0	0%	0	0%	0	0%	0	0%
	KPC	0	0%	0	0%	0	0%	0	0%
	NDM	0	0%	0	0%	0	0%	0	0%
	SPM	0	0%	0	0%	0	0%	0	0%
	VIM	0	0%	0	0%	0	0%	0	0%
	OXA‐48	0	0%	0	0%	0	0%	0	0%
ESBL									
	TEM	20	8%	12	8%	5	8%	3	6%
	CTX‐M	12	5%	12	8%	0	0%	0	0%
	SHV	62	23%	37	24%	16	26%	9	18%
MCR		2	1%	0	0%	2	3%	0	0%
*Pseudomonas* spp.		Samples (n = 262)	%	Samples (n = 153)	%	Samples (n = 61)	%	Samples (n = 48)	%
	Detected	176	67%	120	78%	32	52%	24	50%
Carbapenemases		0	0%	0	0%	0	0%	0	0%
	KPC	0	0%	0	0%	0	0%	0	0%
	NDM	0	0%	0	0%	0	0%	0	0%
	SPM	0	0%	0	0%	0	0%	0	0%
	VIM	0	0%	0	0%	0	0%	0	0%
	OXA‐48	0	0%	0	0%	0	0%	0	0%
ESBL									
	TEM	20	8%	12	8%	5	8%	3	6%
	CTX‐M	1	0%	1	1%	0	0%	0	0%
	SHV	1	0%	0	0%	1	2%	0	0%
MCR		0	0%	0	0%	0	0%	0	0%
*Salmonella* spp.		Samples (n = 418)	%	Samples (n = 307)	%	Samples (n = 61)	%	Samples (n = 50)	%
	Detected	130	31%	111	36%	13	21%	6	12%
Carbapenemases		0	0%	0	0%	0	0%	0	0%
	KPC	0	0%	0	0%	0	0%	0	0%
	NDM	0	0%	0	0%	0	0%	0	0%
	SPM	0	0%	0	0%	0	0%	0	0%
	VIM	0	0%	0	0%	0	0%	0	0%
	OXA‐48	0	0%	0	0%	0	0%	0	0%
ESBL									
	TEM	9	3%	6	4%	2	3%	1	2%
	CTX‐M	19	7%	17	11%	1	2%	1	2%
	SHV	0	0%	0	0%	0	0%	0	0%
MCR		0	0%	0	0%	0	0%	0	0%

*Klebsiella* spp. was isolated in 82.64% (n = 219) of the 265 samples tested, with isolation rates of 81.17% for chicken, 85.25% for pork, and 84% for fish. *Pseudomonas* spp. was isolated from 67.18% (n = 176) of 262 samples, with 78.43% from chicken, 42.46% from pork, and 50% from fish.

### 3.2 Detection of Carbapenemases and MCR Genes

Regarding resistance genes, the *kpc* gene (encoding carbapenemase) was found in 4 *E. coli* isolates out of 350 samples (1.14%). All *kpc*‐positive isolates were from chicken samples (1.49% of chicken isolates). The *mcr‐1* gene was detected in 10 *E. coli* and 2 *Klebsiella* spp. isolates, corresponding to 2.86% and 0.91% detection rates, respectively. Among *E. coli* isolates, nine were from chicken and one from pork. For *Klebsiella* spp., both positive isolates were from pork. MIC testing showed that only one *E. coli* isolate (4 mg/L) and both *Klebsiella* spp. isolates (8 mg/L and >32 mg/L) were phenotypically resistant to colistin.

### 3.3 Detection of ESBL Genes

**FIGURE 2:**
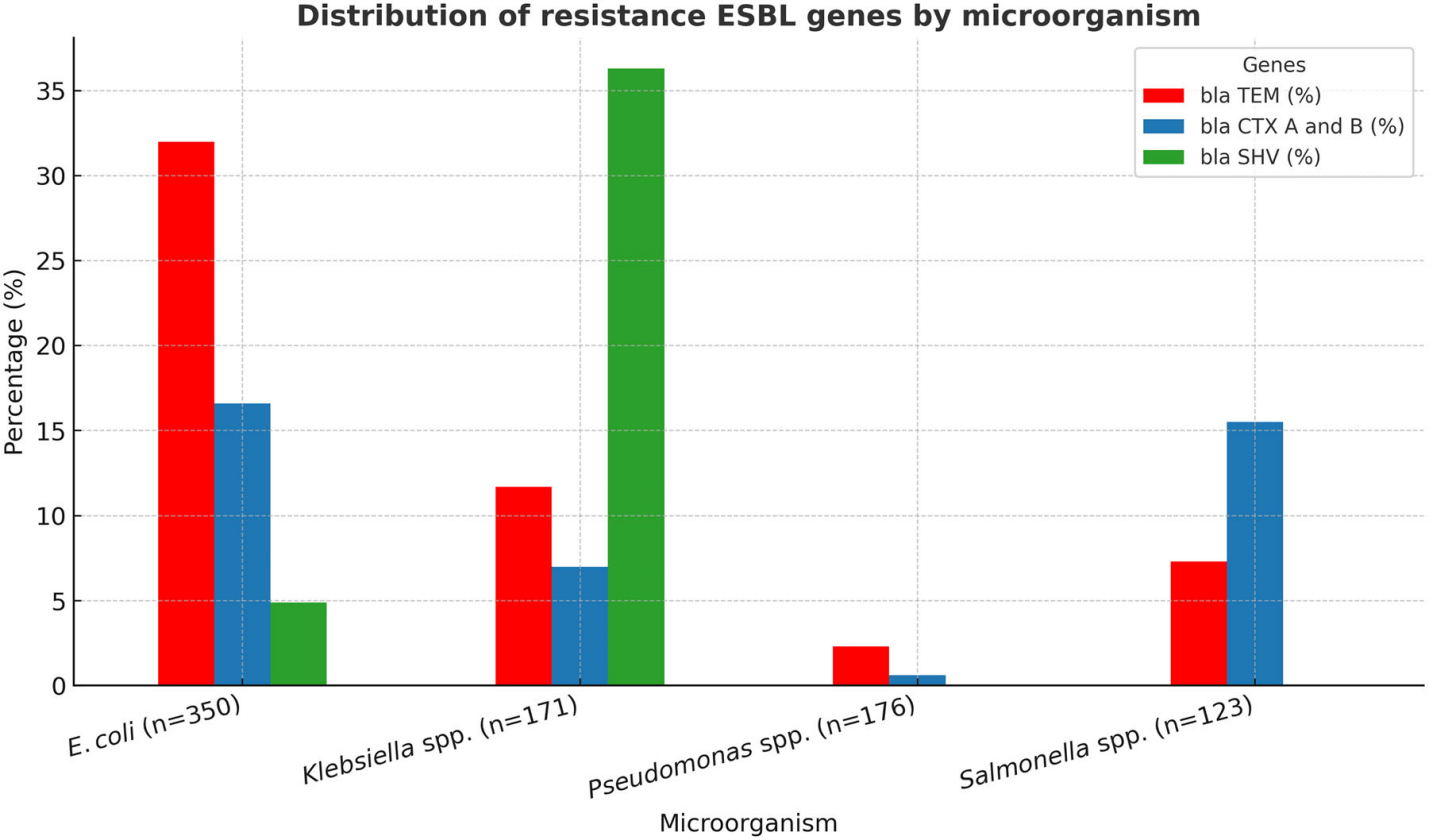
Distribution of extended‐spectrum β‐lactamase (ESBL) resistance genes among different bacterial species recovered from animal samples.

The presence of extended‐spectrum β‐lactamase (ESBL) genes, specifically *bla*‐**TEM**, *bla*‐**CTX‐M**, and *bla*‐**SHV**, was evaluated across different bacterial species (Figure 2). Among *E. coli* isolates (n = 350), *bla*‐**TEM** was the most frequently detected gene, present in 32% of samples, followed by *bla*‐**CTX‐M** (16.6%) and *bla*‐**SHV** (4.9%). In *Klebsiella* spp. (n = 171), *bla*‐**SHV** predominated (36.3%), with lower frequencies observed for *bla*‐**TEM** (11.7%) and *bla*‐**CTX‐M** (7.0%). For *Salmonella* spp. (n = 123), *bla*‐**CTX‐M** was the most prevalent gene, identified in 15.5% of isolates. In contrast, *Pseudomonas* spp. exhibited limited ESBL gene carriage, with *bla*‐**TEM** detected in only 2.3% of the isolates.

### 3.4 Multiple Resistance Genes

A subset of the isolates harbored multiple ESBL genes (Table 2). In *E. coli*, 12.3% (n = 43) of the strains carried two or more resistance genes, with the *bla*‐TEM and *bla*‐CTX‐M combination being the most frequent. Among *Klebsiella* spp., 10.5% (n = 18) of isolates exhibited co‐occurrence of multiple genes, predominantly the trio *bla*‐TEM, *bla*‐CTX‐M, and *bla*‐SHV. In *Salmonella* spp., the prevalence of multiple ESBL genes was lower, detected in only 3.3% of isolates. Notably, no co‐occurrence of ESBL genes was observed in *Pseudomonas* spp.

### 3.5 Antimicrobial Susceptibility Profiles

Resistance patterns to various antibiotic classes were evaluated across the bacterial species. A high prevalence of multidrug resistance (MDR), defined as resistance to three or more antimicrobial classes, was observed: 47.1% in *E. coli*, 62.7% in *Klebsiella* spp., 68.6% in *Pseudomonas* spp., and 83.7% in *Salmonella* spp. (Table 3). Carbapenem resistance was relatively low among *E. coli* (0.9%), *Klebsiella* spp. (2.4%), and *Salmonella* spp. (0.8%), but notably high in *Pseudomonas* spp. (28%). Cephalosporin resistance showed a trend toward higher resistance to second‐generation cefoxitin when compared to third‐ and fourth‐generation agents. Ampicillin resistance was widespread, reaching up to 88.8% in *Klebsiella* spp. Additionally, tetracycline resistance was substantial, particularly in *Salmonella* spp. (82.1%). Resistance to sulfamethoxazole/trimethoprim and ciprofloxacin was also significant, especially among *E. coli* isolates.

**TABLE 3:** Antimicrobial susceptibility profiles of bacterial isolates (*E. coli*, *Klebsiella* spp., *Pseudomonas* spp., and *Salmonella* spp.) recovered from meat samples of animal origin. The table shows the percentage of isolates classified as susceptible (S%), intermediate (I%), or resistant (R%) to a panel of antibiotics, including β‐lactams, aminoglycosides, fluoroquinolones, tetracyclines, and others. Data are summarized per bacterial species and cumulatively in the “Total” column. Dashes (–) indicate tests not performed or not applicable for the given organism‐antibiotic combination.

	E. coli (n = 396)			*Klebsiella* spp. (n = 219)			*Pseudomonas* spp.(n = 176)			Salmonella spp. (n = 130)			Total		
**Antibiotic**	S%	I%	R%	S%	I%	R%	S%	I%	R%	S%	I%	R%	S%	I%	R%
Amoxicillin‐Clavulanate	76	0	24	0	38	62	—	—	—	31	0	69	46	11	43
Ampicillin	56	0	44	0	20	80	19	19	62	27	0	73	32	8	60
Azithromycin	98	0	2	0	88	12	92	0	8	98	0	2	73	21	6
Cefepime	85	0	15	2	93	5	20	66	14	87	1	12	53	35	12
Cefotaxime	84	4	12	2	92	7	0	85	15	54	21	25	44	43	13
Cefoxitin	86	0	14	0	57	43	—	—	—	32	0	68	51	17	32
Ceftazidime	89	4	8	2	93	5	0	95	5	32	16	52	43	44	13
Chloramphenicol	80	0	20	0	90	10	93	0	7	99	0	1	66	21	12
Ciprofloxacin	66	4	30	8	82	10	99	0	1	87	5	8	62	22	17
Gentamicin	81	0	19	0	90	10	71	6	24	95	0	5	62	22	16
Imipenem	99	1	0	20	78	2	0	73	27	100	0	0	61	33	6
Sulfamethoxazole‐trimethoprim	61	0	39	4	64	32	85	0	15	96	0	4	57	15	28
Tetracycline	48	0	52	0	40	60	11	35	54	18	0	82	25	16	58
Tigecycline	100	0	0	0	97	3	—	—	—	100	0	0	71	28	1

These findings should be interpreted in light of some methodological limitations: sampling was uneven across years, animal species, and regions; retail‐based collection may not fully reflect farm‐level resistance; *Klebsiella spp*. and *Pseudomonas spp*. were only included from 2019 onward; susceptibility testing relied on disk diffusion, which may underestimate resistance compared to MIC‐based methods; and the absence of molecular typing (e.g., WGS) limited insights into clonal spread and transmission dynamics.

## 4 Discussion

The data published in 2024 by the European Food Safety Authority (EFSA) and the European Centre for Disease Prevention and Control (ECDC)—covering bacterial isolates from human infections and monitoring of animal production chains—reported findings from samples collected between 2021 and 2022 (European Food Safety et al. 2024). These data are compared with the results of the present study. In the biennium, resistance rates in *Salmonella* isolates from poultry were reported at 55.5% for fluoroquinolones (ciprofloxacin), and 10.1% for isolates from pork. Data for fish were not evaluated. Resistance to third‐generation cephalosporins in *Salmonella* isolates from poultry was reported as 1.4% for cefotaxime and 1.3% for ceftazidime, while in pigs it was 0.9% for both antibiotics. MDR rates—defined as resistance to three or more antimicrobial classes—were 43.6% in poultry and 39.1% in pigs.

Compared to the European data, resistance to fluoroquinolones in *Salmonella* was notably lower in the present study (8.1% overall and 9.6% in poultry samples). Conversely, resistance to third‐generation cephalosporins, particularly ceftazidime, was substantially higher in poultry isolates from Paraná (60.6%) compared to European findings. Carbapenem resistance was absent in the European monitoring of *Salmonella*, whereas this study detected a 0.9% resistance rate to imipenem. Higher resistance rates were also found for ampicillin (69.1% vs. 19.2%), chloramphenicol (7.3% vs. 2.8%), gentamicin (6.5% vs. 1.2%), and tetracycline (82.1% vs. 40.2%) compared to European surveillance (European Food Safety et al. 2024). The only antibiotic with higher resistance in Europe was tigecycline (25%), against no detected resistance in Paraná.

Regarding MDR profiles, the percentage of MDR *Salmonella* strains in Paraná was double that reported in Europe for poultry and more than double for pork. When compared to *Salmonella* isolates from imported poultry assessed by EFSA, resistance rates for ciprofloxacin were similar, though cephalosporin resistance remained lower in Europe. For commensal *E. coli* isolated from poultry, European resistance rates were 1.1% for cefotaxime and ceftazidime, and no resistance to meropenem was observed. In Paraná, resistance to third‐generation cephalosporins was approximately ten times higher, while carbapenem resistance remained comparable. Similar resistance rates were observed for ampicillin and tigecycline, but higher rates for tetracycline, chloramphenicol, and gentamicin. Ciprofloxacin resistance was lower in Paraná (29.4%) compared to Europe (46.7%) (European Food Safety et al. 2024).

For *E. coli* isolates from pork, resistance rates were similar to those reported in Europe, with only a slight increase in ceftazidime resistance in this study. Cortés et al. (2022) analyzed *E. coli* isolated from poultry farms in Spain between 2019 and 2021, reporting resistance rates of 66% for trimethoprim‐sulfamethoxazole, 35% for amoxicillin, 9% for cefotaxime, and 38% for tetracycline (Cortes et al. 2022). Our findings showed similar rates for cefotaxime but higher rates for tetracycline and amoxicillin‐clavulanate.

Zhao et al. (2024) conducted a systematic review covering 2000–2019 in low‐ and middle‐income countries. They reported high resistance rates in poultry isolates for tetracyclines (59%), ampicillin (57%), sulfamethoxazole‐trimethoprim (45%), chloramphenicol (35%), ciprofloxacin (30%), gentamicin (28%), and cefotaxime (33%) (Zhao et al. 2024). Our data were consistent with those for tetracycline (58.6%), ampicillin (49.4%), and sulfamethoxazole‐trimethoprim (43.8%). However, we found lower resistance rates for chloramphenicol, ciprofloxacin, and gentamicin. Resistance rates to third‐generation cephalosporins varied, with lower resistance observed for ceftazidime and cefoxitin compared to global cefotaxime rates.

In *Salmonella*, higher resistance rates were found in Paraná for tetracycline (82.1%), ampicillin (72.4%), and cephalosporins (53.7% for ceftazidime and 28.5% for ceftriaxone), while lower resistance rates were observed for sulfamethoxazole‐trimethoprim, chloramphenicol, ciprofloxacin, and gentamicin. Maciel et al. (2019) evaluated *Salmonella* isolates from various animal products in Brazil in 2013, finding 38% resistance to at least one antibiotic and 55% MDR. In comparison, 89.4% of *Salmonella* isolates in our study were resistant to at least one antibiotic, and 83.7% exhibited MDR. Resistance patterns were broadly comparable for ampicillin, tetracycline, and amoxicillin‐clavulanate, although gentamicin resistance was significantly lower (7.3% vs. 45%) (Maciel et al. 2019).

Lauteri et al. (2022) reported on *Salmonella* isolated from pig slaughterhouses in central‐northern Italy, finding 100% resistance to at least one antimicrobial, 87% resistance to tetracycline, 55% to ampicillin, and 27.8% MDR. MDR rates in our study (83.7%) were substantially higher. Antimicrobial‐specific resistance rates were broadly comparable for ampicillin and tetracycline but diverged markedly for gentamicin and amoxicillin‐clavulanate (Lauteri et al. 2022).

To compare resistance found in food isolates to human infections, we used data from 2017 to 2024 reported by the Online Notification System of Hospital Infections (SONIH) from hospitals in Paraná (https://www.saude.pr.gov.br/Pagina/Boletins‐Sonih). Resistance to fourth‐generation cephalosporins or the presence of ESBL genes in human *E. coli* and *K. pneumoniae* isolates was around 28%–29%, lower than the 40% detection rate in food isolates. Conversely, carbapenem resistance was higher in clinical isolates. *P. aeruginosa* isolates from human infections exhibited 26% resistance to carbapenems, closely matching the 28% imipenem resistance found in foodborne *Pseudomonas* isolates.

Plasmid‐mediated colistin resistance (via the *mcr‐1* gene) was first described in enterobacteria in China and has since been reported worldwide (Du et al. 2016). It has been linked to imported foods, urban rivers, and travelers. MCR‐1‐positive isolates frequently harbor other resistance mechanisms, such as carbapenemases and ESBLs (Mediavilla et al. 2016). In 2016, a silent dissemination of *mcr‐1* in South America among food‐producing animals, companion animals, and the environment was described, predominantly in *E. coli* co‐harboring CTX‐M genes—findings consistent with our observations in Paraná (Rau et al. 2020). The timeline of *mcr‐1* detection in poultry samples in Paraná—three isolates in 2017, four in 2018, and two in 2019—coincides with the Brazilian Ministry of Agriculture (MAPA) Ordinance No. 45 of 2016 prohibiting colistin use as a growth promoter, with remaining stocks allowed until late 2017. Judicial intervention subsequently enforced complete suspension. In pork samples, positive *mcr‐1* isolates were detected only after monitoring began in 2021, with one case in 2022 and two in 2023 (Menck‐Costa et al. 2023).

Despite the comprehensive scope and temporal breadth of the dataset, this study has several limitations that must be acknowledged. First, the sampling was not uniformly distributed across all years, animal species, or regions within Paraná, potentially introducing selection bias and limiting generalizability. The inclusion of *Klebsiella* spp. and *Pseudomonas* spp. only from 2019 onward also restricts temporal comparisons with earlier data. Additionally, the study relied on retail‐level sampling, which, while useful for assessing consumer exposure, may not fully capture resistance profiles present at the farm or processing levels. The exclusion of molecular typing techniques, such as whole‐genome sequencing (WGS), limited the ability to determine clonal relationships and track transmission dynamics of resistant strains. Lastly, the reliance on the disk diffusion method for susceptibility testing, although standardized, may underestimate resistance to certain antimicrobials compared to more sensitive MIC‐based approaches. Most critically, the absence of whole‐genome sequencing (WGS) and plasmid typing limited the molecular epidemiology of this study: we were unable to determine clonal relationships among isolates, identify specific plasmid backbones carrying resistance genes, or track horizontal gene transfer events that could explain the spread of ESBLs, carbapenemases, and mcr‐1. This gap reduces the capacity to link foodborne isolates to clinical strains and to evaluate dissemination pathways.

While international comparisons with EFSA and other global monitoring programs help contextualize the magnitude of resistance, the Brazilian scenario carries specific challenges that amplify the public health risks. In Paraná, intensive poultry and pork production is closely tied to high antimicrobial consumption, often with limited oversight at the farm level. Despite regulatory advances, such as the 2016 ban on colistin as a growth promoter and the establishment of PAN‐BR and PAN‐VISA, enforcement remains uneven, and off‐label or prophylactic use of critically important antimicrobials persists. The elevated resistance rates observed here—particularly for third‐generation cephalosporins and tetracyclines—mirror these gaps and highlight the vulnerability of Brazilian production systems to resistance dissemination. For public health, this translates into a greater risk of resistant bacteria entering the food chain and ultimately the hospital setting, where ESBL‐ and carbapenemase‐producing *Enterobacterales* are already a growing problem. Strengthening farm‐level biosecurity, improving veterinary prescription practices, and reinforcing surveillance programs like PAMVet PR are crucial steps to mitigate these risks and align Brazil more closely with international One Health strategies.

This study provides robust evidence of widespread antimicrobial resistance among foodborne bacterial isolates obtained from meat samples in the State of Paraná, Brazil, between 2017 and 2023. The detection of ESBL genes, the presence of multidrug‐resistant profiles, and the identification of carbapenem‐ and colistin‐resistant strains — including mcr‐1‐ and kpc‐positive isolates — highlight the urgent need for continued surveillance and mitigation strategies. Resistance levels observed in *E. coli*, *Klebsiella* spp., *Salmonella* spp., and *Pseudomonas* spp. were in many cases higher than those reported in European and global surveillance efforts, particularly regarding third‐generation cephalosporins, tetracyclines, and ampicillin. These findings underscore the critical role of veterinary antibiotic usage as a driver of resistance dissemination into the human food chain.

Given Brazil's prominent role as a leading global exporter of animal protein, these results reinforce the importance of expanding national programs such as PAMVet PR. Coordinated efforts among health, agriculture, and environmental sectors, aligned with the One Health framework, are essential to curbing the spread of AMR. This study contributes valuable epidemiological data to inform public health policies and supports the development of targeted interventions to limit the use of critically important antimicrobials in animal production, enhance regulatory oversight, and protect both animal and human health.

## Author Contributions

**André Schenkel Dedecek**: conceptualization, writing – original draft, investigation. **Lavinia Arend**: investigation, methodology, data curation. **Margareth Leonor Penkal**: investigation, writing – original draft. **Salesia Maria Prodocimo Moscardi**: writing – original draft. **Felipe Francisco Tuon**: formal analysis, writing – original draft, writing – review and editing.

## Funding

The authors have nothing to report.

## Ethics Statement

The authors have nothing to report.

## Conflicts of Interest

The authors declare no conflicts of interest.

## Data Availability

The datasets used and/or analyzed during the current study are available from the corresponding author on reasonable request.
